# Whole-exome sequencing identifies matrisomal gene associations in monogenic cerebral small vessel disease

**DOI:** 10.1007/s00415-026-14018-2

**Published:** 2026-07-21

**Authors:** Solomon. K. Guyler, Mohammed. M. Alfayyadh, Neven Maksemous, Rodney. A. Lea, Robert. A. Smith, Heidi. G. Sutherland, Lyn.R. Griffiths

**Affiliations:** 1 Genomics Research Centre, Centre for Genomics and Personalised Health, School of Biomedical Sciences, Queensland University of Technology (QUT), Brisbane, QLD Australia; 2 Central Analytical Research Facility (CARF), Queensland University of Technology (QUT), Brisbane, Australia

**Keywords:** Cerebral small vessel disease, Leukoencephalopathy, Burden testing, CADASIL, Stroke, Dementia

## Abstract

Cerebral small vessel diseases (CSVDs) are a group of disorders affecting the small arteries, veins, and capillaries supplying the white matter and deep grey matter structures. They are the most common form of cerebrovascular disease, accounting for approximately half of vascular dementia cases and 20% of stroke incidence. Whilst genetic testing is a routine diagnostic tool for monogenic CSVDs, less than 20% of patients have a causal variant in known CSVD genes. We performed whole exome sequencing on 117 patients suspected of monogenic CSVD who previously tested negative for pathogenic variants in seven well-characterised CSVD genes (*NOTCH3, HTRA1, COL4A1, COL4A2, TREX1, GLA,* and *FOXC1).* Targeted analysis was conducted on known and associated CSVD genes, as well as candidate genes which cause conditions with overlapping symptomology to CSVD. Burden analysis focussing on rare, functional variants was used to identify novel associations when compared against a cohort of 1035 non-neurological controls. We identified 18 suspected disease-causing variants across nine CSVD-associated genes and a significant burden of both rare and rare, likely disease-causing heterozygous variants in *ABCC6*. Two genes from stroke and neurodegenerative disease gene panels also possessed a significant burden of rare, likely disease-causing variants, *MYH11* (adjusted *P* = 1 × 10^–2^) and *NOTCH1* (adjusted *P* = 1 × 10^–2^). We further identified novel associations for seven genes (*COL7A1, HMCN1, LAMA1, MMP9, TENM4, TNC, TTN*) with monogenic CSVD in this cohort. Our findings implicate several genes as potentially causal of monogenic CSVD, highlighting the need for more extensive genetic screening in suspected CSVD cases, and functional characterisation of implicated variants to determine their mechanistic role in CSVD pathogenesis.

**Supplementary Information:**

The online version contains supplementary material available at 10.1007/s00415-026-14018-2.

## Introduction

Cerebral small vessel disease (CSVD) is a chronic, progressive condition that affects the small blood vessels of the brain. It is the most common form of cerebrovascular disease and includes a group of disorders that collectively represent the most prevalent monogenic cause of stroke, typically presenting with deep lacunar infarcts and widespread white matter changes [1]. The most common monogenic forms of CSVD are cerebral autosomal dominant arteriopathy with subcortical infarcts and leukoencephalopathy (CADASIL), caused by pathogenic variants in *NOTCH3*; CADASIL2, caused by heterozygous variants in *HTRA1*; Gould syndrome, resulting from variants in *COL4A1/2*; and Fabry disease, caused by variants in *GLA* [2]. Genetic testing of CSVD genes is routine when a monogenic small vessel disease is suspected; however, such screening only detects causal mutations in ~ 20% of patients, suggesting that there are as yet unidentified genes contributing to monogenic CSVD [3, 4].

**Fig. 1:**
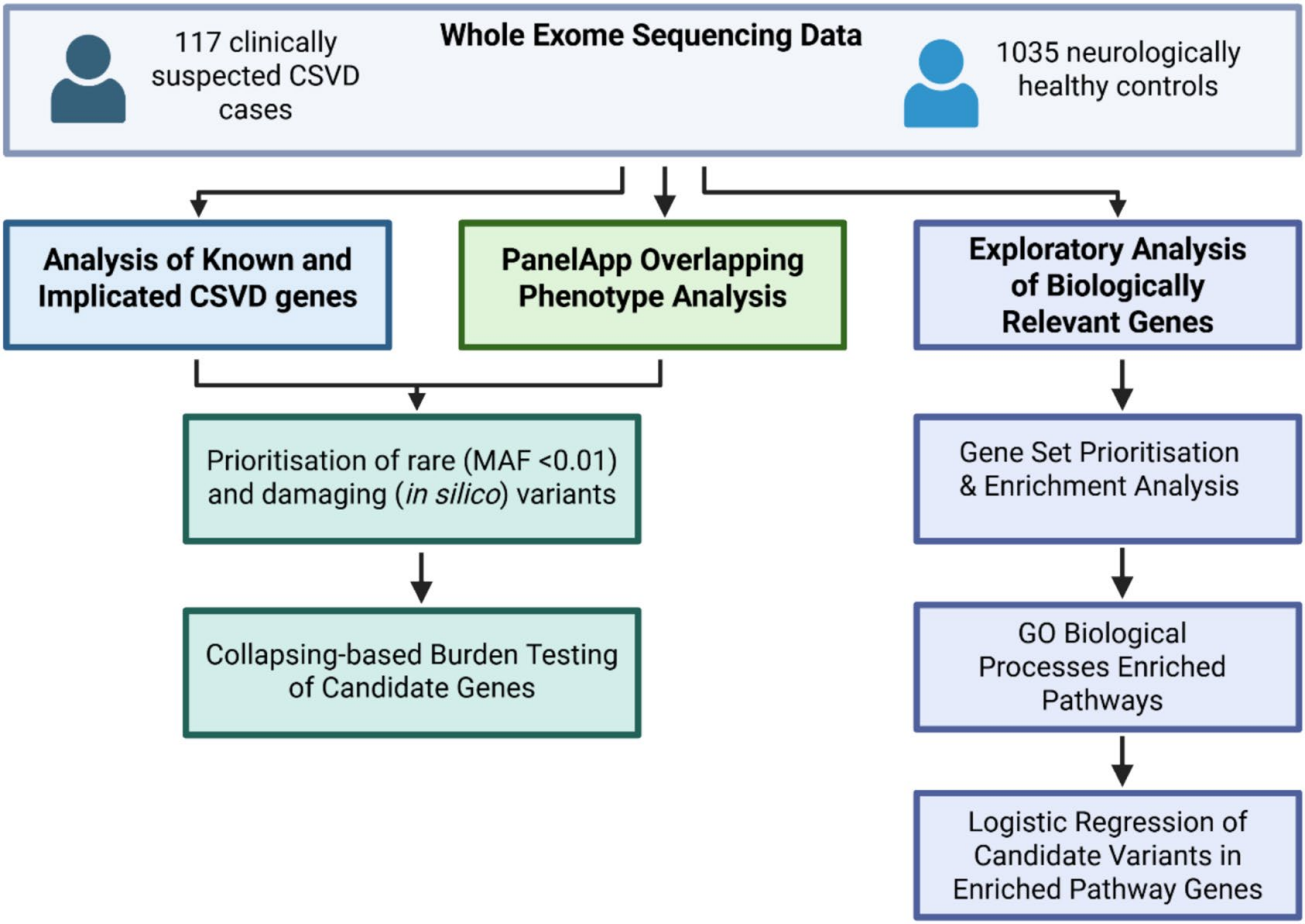
Study workflow. Incorporating WES data from our patient cohort and UKBB neurologically healthy controls, we first performed a targeted investigation of causal CSVD genes and genes associated with CSVD or cerebrovascular pathology. We then explored genes causative of alternate disorders with phenotypic overlap to monogenic CSVDs. Finally, we performed an exploratory analysis using gene set prioritisation and enrichment analyses to identify novel genetic associations with CSVD

Whole-exome sequencing (WES) is a well-characterised approach in the detection of novel associations between genes and Mendelian diseases and has been used previously to identify novel genetic causes of CSVD [5, 6]. The Genomics Research Centre (GRC) has been conducting diagnostic genetic testing for CADASIL, the most common monogenic CVSD, since 1997. However, only ~ 12% of suspected patients are identified with a causal variant in *NOTCH3* [3, 7]. The expansion of diagnostic testing to include further monogenic CSVD genes (*HTRA1, COL4A1, COL4A2, GLA, TREX1, FOXC1*) resulted in an additional ~ 14% improvement in the detection of likely disease-causing variants, however, the majority of suspected patients remain without an identified causal mutation [8]. In this study, we performed WES on a cohort of 117 patients clinically suspected of monogenic CSVD and novel associations with CSVD were investigated using several bioinformatic methods and gene-based burden testing (Fig. 1).

## Methods

### Patient cohort

The study cohort included 117 patients referred to the Genomics Research Centre (GRC) (Queensland University of Technology, Brisbane, Australia) for molecular genetic testing for monogenic CSVD according to National Association for Testing Authority, Australasia (NATA) standards. 1281 patients were referred to the GRC by accredited neurologists within Australia and New Zealand between 1997 and 2024 and participants in this study selected based on the following inclusion criteria: (1) Clinical suspicion of CADASIL or other monogenic CSVD with clinical signs and symptoms consistent with monogenic CSVD, including family history of cerebral vascular disease, recurrent subcortical ischaemic stroke, evidence of vascular leukoencephalopathy or dementia, cognitive decline, migraine, or gait disturbance; (2) an absence of vascular risk factors including hypertension and hyperlipidaemia; and (3) negative for pathogenic variants within a targeted CSVD gene panel, which included *NOTCH3, HTRA1, COL4A1, COL4A2, TREX1, FOXC1,* and *GLA*. Clinical and demographic characteristics of the study cohort and control subjects are summarised in Table S1.

### Whole-exome sequencing

DNA was extracted from peripheral blood using the QIAGEN QIACUBE™ according to the manufacturer’s instructions and quantified using the Nanodrop 8000 Spectrophotometer (Thermo Fisher Scientific). WES was performed using the Ion AmpliSeq Exome RDY-kits for library preparation according to the manufacturer’s instructions (MAN0010084). Library quantification was performed using an Invitrogen Qubit 3 Fluorometer (Thermo Fisher Scientific) for equimolar pooling prior to sequencing. Template preparation, enrichment and chip loading were performed using the Ion 550™ Kit-Chef (Cat. Number A34541) and 550 Chips on the Ion Chef. Sequencing was performed using the Ion S5 + platform with sequences aligned to human genome 38 (Hg38) and variant calling performed using PathVAR, a customisable NGS pipeline for whole exome/genome sequencing analysis and annotation [9]. Coverage and mapping parameters showed an average coverage of 118X with a mean number of reads of 39,275,917. An average of 96% of reads were on target and the average > 20X coverage was 89%.

### Control cohort generation

The control cohort used in this study was derived from the UK Biobank whole-genome sequencing (WGS) resource, which includes genetic, biochemical, and imaging data for over 500,000 participants [10]. From this cohort, we obtained data for a subset of 1035 aged individuals (mean age = 53; SD = 7.3; 518 males, 517 females) without any history of neurological or cerebrovascular disease, serving as neurologically healthy controls. To equate the control dataset to the CSVD cohort, whole exome data for these individuals were extracted from WGS base alignment mapping (BAM) files using the view function in the BCFTools package [11].

### Estimation of relatedness and linkage

Underlying population structure and relatedness were assessed through principal component analysis (PCA) of genotypes shared between our case and control cohort and the 1000 Genome Project Phase 3 data [12]. 12,122 single-nucleotide polymorphisms (SNPs) were selected after removing all SNPs with a minor allele frequency (MAF) of ≤ 0.05. SNPs in linkage disequilibrium were pruned using PLINK1.9 software with a window size of 50 bp, a window shift of 5 bp, and a variant inflation factor of 0.1 [13]. Filtered samples were then used to perform a PCA using PLINK1.9. Related individuals were identified using pairwise identity-by-descent (IBD) distances, and kinship values were generated and extracted with samples with an IBD > 0.1 excluded from downstream analysis. Of the 117 participants in the case cohort, 94.8% were assigned to European ancestry (*n* = 111), 2.56% to South Asian (*n* = 3), and 2.56% to East Asian (*n* = 3). All control samples (*n* = 1035) from the UKBB were assigned European ancestry and included in downstream burden analysis.

### Variant filtering

Variants were filtered based on coverage and allele ratio with a minimum coverage of 20 × and an allele ratio of 35% to 65% used for heterozygous variants and > 85% used for homozygous variants. Variants predicted to alter protein function, including frameshift, non-synonymous, stop-gain, and stop-loss variants, were retained. Population frequency filtering was applied using gnomAD v4.1.0, with variants observed at an MAF ≤ 0.01 in non-Finnish Europeans retained for downstream analysis. Functional impact was assessed using in silico prediction tools, including Polymorphism Phenotyping v2 (PolyPhen-2), Sorting Intolerant From Tolerant (SIFT4G), REVEL, and Combined Annotation Dependent Depletion (CADD v1.7) [14–17]. Variants classified as deleterious by at least two tools using score guidelines in accordance with American College of Medical Genetics (ACMG) guidelines were retained as likely damaging for burden testing [18]. Variants were further assessed using ClinVar with variants classified as benign or likely benign excluded as not disease-causing.

### Burden testing

Burden testing was performed using a gene-based collapsing framework. For each gene in the lists outlined below, individuals carrying at least one candidate variant were compared between cases and controls. Statistical testing was performed for known and associated CSVD genes and PanelApp candidate genes using Fisher’s exact test, implemented in R, whilst logistic regression using the glmnet package in R was used for the exploratory analysis, with age and sex included as covariates in the regression model [19]. A nominal significance threshold of *P* < 0.05 was applied, and correction for multiple testing was performed using the Benjamin–Hochberg false discovery rate (FDR) [20]. Genes with an adjusted *P* < 0.05 were considered statistically significant.

### Known and implicated CSVD gene analysis

**Fig. 2:**
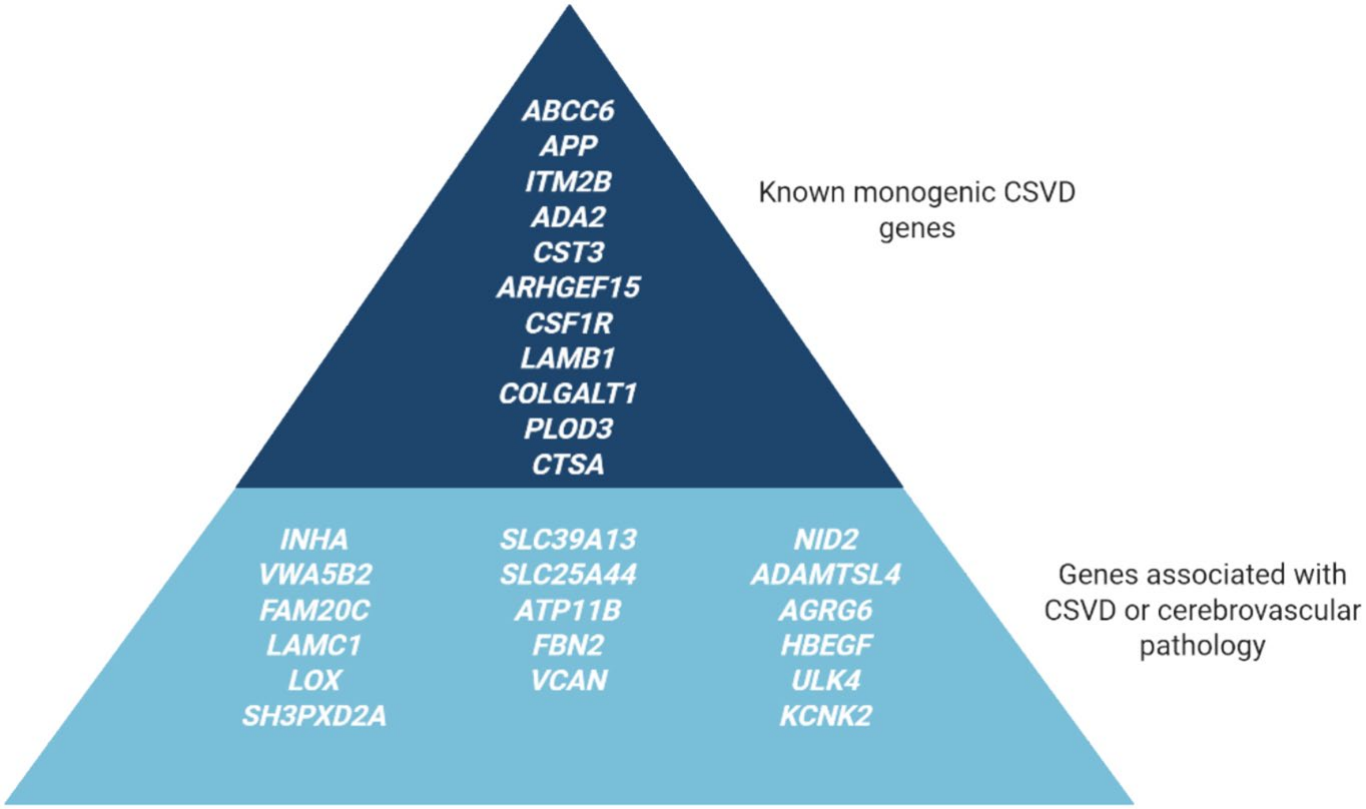
Genes selected for rare variant analysis*.* Genes were stratified into two tiers based on strength of evidence. Known monogenic CSVD genes (dark blue) represent established causal variants for distinct hereditary CSVDs, whilst genes associated with CSVD or broader cerebrovascular pathology (light blue) were implicated through burden testing, GWAS, TWAS, or functional studies

Analysis was performed on known and associated CSVD genes and genes implicated in cerebrovascular pathology (Fig. 2) [5, 21–24]. Candidate rare, likely disease-causing variants were identified following filtering and classification steps described above.

### PanelApp gene set analysis

Targeted analysis was conducted using gene panels with potential overlap to CSVD which were curated in PanelApp Australia, including Stroke (v1.16), Cerebral vascular malformations (v1.0), Leukodystrophy–adult-onset (v0.147), Neurodegenerative disease–adult-onset (v6.118), and Cerebral amyloid angiopathy (v1.1) (Table S2) [25].

### Gene set enrichment analysis

A gene set was curated where genes containing at least one rare, likely disease-causing variant were collated. This gene set was prioritised using ToppGene Suite (default parameters), with known CSVD genes (*NOTCH3, HTRA1, COL4A1, COL4A2, GLA, TREX1, CTSA, FOXC1, ABCC6, COLGALT1, APP, CST3, ITM2B, ADA2*) used as the training set [26]. The ranked list of genes was then subjected to gene set enrichment analysis (GSEA) using WebGestalt (default parameters), focussing on Gene Ontology Biological Processes to identify pathways relevant to CSVD pathogenesis [27]. Genes identified in significantly enriched pathways from GSEA were obtained, resulting in a list of candidate genes for association analysis using logistic regression (Table S3). Genes used within the training set for gene set prioritisation were excluded from this analysis due to their established role in CSVD. Significantly associated genes were retained for further evaluation.

### Gene expression analysis

Gene expression data were obtained from the Genotype-Tissue Expression (GTEx) portal and the CELLxGENE Discover corpus [28, 29]. Expression was assessed across brain regions and vascular cell types. Aggregated expression across tissues was calculated, and genes were assessed by scaled expression and percentage tissue composition.

## Results

### Analysis of known and recently implicated CSVD genes

117 patients suspected of monogenic CSVD underwent WES to identify potential causes of disease. Twenty-eight genes previously identified as causal or associated with monogenic CSVD were assessed for causal variants in this cohort (Fig. 2). 75 rare variants were identified across 19 genes, with several occurring in multiple individuals (Table S4). No variants were detected in *FAM20C*, *CST3*, *ITM2B, LOX, AGRG6, KCNK2, SLC39A13, SLC25A44,* or *CTSA*.

When restricting to variants predicted to be damaging via in silico tools, 18 rare, likely disease-causing variants were identified across nine genes in the CSVD cohort: *ABCC6, LAMB1, APP, COLGALT1 LAMC1, EFEMP1, FBN2, SH3PXD2A,* and *VCAN* (Table 1). Of the 18 variants identified, 2 had previously been classified as pathogenic, 10 as variants of uncertain significance (VUS), and 6 had not previously been annotated in ClinVar. Five heterozygous variants were identified in *ABCC6*, including two pathogenic variants and three classified as variants of uncertain significance (VUS).

**Table 1:** Rare, likely disease-causing variants identified in known and implicated CSVD genes

Gene	Chromosome position	Variant	Patient	Minor allele frequency	ClinVar classification	CADD	Poly Phen	SIFT	REVEL
*ABCC6*	chr16:16,163,078	c.3421C > T p.Arg1141Ter	DGR545	0.001943	Pathogenic	42	N/A	N/A	–
*ABCC6*	chr16:16,202,006	c.1171A > G p.Arg391Gly	DGR25	0.007767	Pathogenic/VUS/Likely Benign	22.9	1	0.002	0.847
*ABCC6*	chr16:16,182,476	c.2183C > G p.Ala728Gly	DGR569	0.00001695	VUS	23.4	0.95	0.004	0.739
*ABCC6*	chr16:16,169,823	c.2818C > T p.Arg940Cys	DGR271	8.68E-07	VUS	23.9	0.591	0	0.612
*ABCC6*	chr16:16,157,692	c.3853G > A p.Val1285Met	DGR363	0.00003305	VUS	22	0.591	0.004	0.477
*APP*	chr21:26,000,072	c.976G > A p.Gly326Ser	DGR349	0.00002288	VUS	27.7	0.979	0	0.591
*APP*	Chr21:26,000,019	c.1027A > C p.Ser343Arg	DGR474	8.475E-07	N/A	25.1	0.741	0	0.334
*LAMB1*	chr7:107,975,088	c.1380C > A p.Cys460Ter	DGR24	0.000001707	N/A	39	N/A	N/A	–
*LAMB1*	chr7:107,980,808	c.680 T > C p.Leu227Ser	DGR330	0.001144	VUS/Likely Benign	24.2	0.702	0.001	0.682
*LAMB1*	chr7:107,959,319	c.2620G > T p.Asp874Tyr	DGR556	N/A	N/A	23.7	0.982	0.01	0.407
*LAMC1*	chr1:183,127,313	c.3032G > A p.Arg1011His	DGR524	0.006805	N/A	25.6	0.987	0.006	0.592
*LAMC1*	chr1:183,118,125	c.1969C > A p.Arg657Ser	DGR531	0.0001153	N/A	28.1	1	0	0.625
*LAMC1*	chr1:183,110,594	c.961C > T p.Pro321Ser	DGR558	0.001071	N/A	25.7	0.975	0.018	0.374
*COLGALT1*	chr19:17,692,146	c.1762C > T p.Arg588Cys	DGR75	0.0003288	VUS	27.2	1	0.011	0.731
*EFEMP1*	chr2:55,881,684	c.568 T > C p.Cys190Arg	DGR24	0.000005085	VUS	27.8	0.999	0	0.974
*FBN2*	chr5:128,263,603	c.8014A > G p.Thr2672Ala	DGR538	8.457E-7	VUS	27.7	0.996	0	0.877
*SH3PXD2A*	chr10:103,735,778	c.260G > A p.Arg87Gln	DGR342	0.000005932	VUS	26.6	0.96	0	0.293
*VCAN*	chr5:83,512,306	c.952G > T p.Gly318Cys	DGR27	0.00002882	VUS	29.5	0.999	0	0.372

The chromosomal assembly used in this analysis was human genome 38 (hg38). Minor allele frequencies were obtained from the GnomAD v4.1.0 non-Finnish European population. Transcript identifiers: LAMB1 (NM_002291.3), APP (NM_000484.4), ABCC6 (NM_001171.6), LAMC1 (NM_002293.3), COLGALT1 (NM_024656.3), EFEMP1 (NM_001039348.3), FBN2 (NM_001999.4), SH3PXD2A (NM_001394015.1), and VCAN (NM_004385.5)

Principal component analysis was performed for ancestry inference, and six cases were subsequently excluded due to non-European ancestry, with 111 CSVD cases retained for burden analyses. Burden testing of identified genes identified a significant association in the CSVD cohort for *ABCC6*, both when considering rare variants (adjusted *P* = 9.42 × 10⁻⁶) and rare, likely damaging variants (adjusted *P* = 0.011) (Table 2). A significant association was also observed for rare *FBN2* variants which were enriched in the control cohort (adjusted *P* = 0.041, OR = 0.30), indicating a lower frequency of *FBN2* variants amongst individuals with CSVD.

**Table 2:** Fisher’s exact test comparing the burden of rare variants and rare likely disease-causing variants in known and implicated CSVD genes

Gene	Rare case count	Rare control count	P value (adjusted)	Odds ratio	Del case count	Del control count	P value (adjusted)	Odds ratio
*ABCC6*, n (%)	5 (4.4)	0	7.85E-07 (1.57E-05)	Inf	3 (2.7)	0	0.00089 (0.018)	Inf
*LAMB1*, n (%)	5 (4.4)	25 (2.4)	0.20 (0.74)	1.90	3 (2.7)	5 (0.5)	0.03 (0.3)	5.71
*LAMC1*, n (%)	7 (6.2)	46 (4.4)	0.34 (0.8)	1.45	3 (2.7)	10 (1.0)	0.12 (0.56)	2.84
*APP*, n (%)	2 (1.8)	8 (0.8)	0.25 (0.74)	2.35	2 (1.8)	5 (0.5)	0.14 (0.56)	3.77
*SH3PXD2A*, n (%)	1	5	0.47 (1)	1.77	1	0	0.1 (0.56)	Inf
*COLGALT1*, n (%)	6 (5.3)	32 (3.1)	0.26 (0.74)	1.79	1 (0.9)	3 (0.3)	0.34 (0.76)	3.12
*EFEMP1*, n (%)	1	7	0.58 (0.97)	1.27	1	2	0.28 (0.76)	4.44
*FBN2*, n (%)	5	133	0.0041 (0.041)	0.3	2	8	0.27 (0.76)	2.23
*VCAN*, n (%)	7	85	0.48 (1)	0.71	1	13	1	0.68
*ARHGEF15*, n (%)	6 (5.3)	33 (3.2)	0.26 (0.74)	1.73	0	3 (0.3)	1	0
*CSF1R*, n (%)	5 (4.4)	50 (4.8)	1	0.93	0	27 (2.6)	1	0
*PLOD3*, n (%)	4 (3.5)	41 (4.0)	1	0.91	0	11 (1.1)	1	0
*VWA5B2*, n (%)	3 (2.7)	25 (2.4)	0.75 (1)	1.12	0	4 (0.4)	1	0
*ATP11B*, n (%)	2 (1.8)	19 (1.8)	1	0.98	0	2 (0.2)	1	0
*INHA*, n (%)	1 (0.9)	19 (1.8)	0.71 (1)	0.49	0	3 (0.3)	1	0
*ADA2*, n (%)	2 (1.8)	14 (1.4)	0.66 (1)	1.34	0	1 (0.1)	1	0
*ADAMTSL4*, n (%)	3	55	0.26 (0.74)	0.47	0	6	1	0
*HBEGF*, n (%)	1	9	1	0.98	0	3	1	0
*NID2*, n (%)	18	157	0.89 (1)	1.02	0	22	1	0
*ULK4*, n (%)	12	79	0.36 (0.8)	1.38	0	2	1	0

Adjusted *P* values were calculated using Benjamini–Hochberg False Discovery Rate. Del = Likely Deleterious. Variants classified as deleterious by at least two tools using score guidelines in accordance with American College of Medical Genetics (ACMG) guidelines were retained as candidate variants for burden testing

### Investigation of candidate genes from PanelApp Australia gene panels

Genes included in neurodegenerative disease, stroke, cerebrovascular malformation, or leukodystrophy panels obtained from the PanelApp Australia resource were analysed. Variants in genes previously implicated in CSVD were excluded from this analysis. Rare, likely disease-causing variants were identified in 35 of 111 individuals (31.5%), all of which were heterozygous. In total, 34 unique variants were observed across 24 genes, including 30 missense variants and four nonsense variants introducing premature stop codons (Table S5). Four patients were identified with pathogenic variants, including three patients with a pathogenic variant in *SQSTM1*, causative of frontotemporal dementia with amyotrophic lateral sclerosis, and one patient carrying a nonsense variant in *ANGPTL6* which has been shown to cause familial intracranial aneurysm (Table 3).

**Table 3:** Disease-causing variants identified in genes associated with alternate disorders

Patient	Chromosome position	Gene	Variant	Minor allele frequency	ClinVar classification	CADD	Poly Phen	SIFT	REVEL
DGR350 DGR527	chr5:179,836,445	*SQSTM1*	c.1175C > T p.Pro392Leu	0.001674	Pathogenic/Likely pathogenic/VUS	24.7	0.3	0.07	0.818
DGR565	chr5:179,823,038	*SQSTM1*	c.286C > T p.Arg96Ter	0.00001356	Pathogenic	44	N/A	N/A	–
DGR524	chr19:10,093,793	*ANGPTL6*	c.851C > G p.Ser284Ter	0.0007635	N/A	36	N/A	N/A	–

The chromosomal assembly used in this analysis was human genome 38 (hg38). Minor allele frequencies were obtained from the GnomAD v4.1.0 non-Finnish European population. Transcript identifiers: SQSTM1 (NM_003900.5), ANGPTL6 (NM_031917.3). VUS variant of uncertain significance

**Fig. 3:**
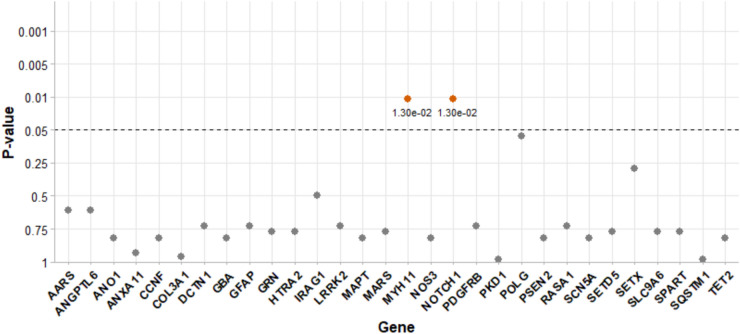
Gene-based burden testing results from genes included in candidate PanelApp Australia gene panels. *P* values shown have been adjusted using the Benjamini–Hochberg False Discovery Rate Method. The dotted line indicates the statistical significance threshold of *P* = 0.05

Burden testing was performed on genes containing at least one rare, likely disease-causing variant. Significant associations with the CSVD cohort were identified for two genes, *MYH11* and *NOTCH1,* compared with controls (Fig. 3). Despite there being nine times as many controls as cases, no candidate variants in these genes were detected in the controls compared with three variants in each gene in the case cohort (Table 4). Notably, likely disease-causing variants in each of these genes were also identified in patients who had previously been excluded from burden analysis due to non-European ancestry (Table S6). Comprehensive details of burden testing results are available in Table S7.

**Table 4:** Disease-causing variants identified in significant genes from PanelApp gene panels with phenotypic overlap to CSVD

Patient	Chromosome position	Gene	Variant	Minor allele frequency	ClinVar classification	CADD	Poly Phen	SIFT	REVEL
DGR22	chr16:15,718,337	*MYH11*	c.5294G > A p.Arg1765Gln	0.0002449	Likely Pathogenic/VUS/Likely Benign	29.9	0.982	0.033	0.864
DGR540	chr16:15,720,973	*MYH11*	c.4678G > T p.Asp1560Tyr	–	–	31	1	0	0.833
DGR278	chr16:15,778,801	*MYH11*	c.790G > A p.Val264Met	0.00015	VUS	22.8	0.857	0.003	0.717
DGR538	chr9:136,508,893	*NOTCH1*	c.3148G > A p.Gly1050Ser	0.000006105	VUS	26.9	1	0	0.858
DGR32	chr9:136,506,782	*NOTCH1*	c.3835C > T p.Arg1279Cys	0.0009145	VUS/Likely Benign/Benign	29.7	0.841	0.063	0.436
DGR307	chr9:136,510,659	*NOTCH1*	c.2734C > T p.Arg912Trp	0.003148	VUS/Likely Benign/Benign	25.4	0.85	0.017	0.402

The chromosomal assembly used in this analysis was human genome 38 (hg38). Minor allele frequencies were obtained from the GnomAD v4.1.0 non-Finnish European population. Transcript identifiers: MYH11 (ENST00000396324.7), NOTCH1 (NM_017617.5). VUS variant of uncertain significance

### Identification of monogenic CSVD gene associations

To identify novel associations with CSVD, 5,527 rare, likely disease-causing variants identified via WES across 3,419 unique genes were ranked by biological and functional similarity to established CSVD genes. Gene set enrichment analysis (GSEA) focussing on GO: biological processes identified ten significantly enriched pathways, with endoderm development and morphogenesis of a branching structure identified as the most significant (Table S8). A total of 221 unique genes were identified from GSEA enriched pathways and retained for burden analyses, excluding genes previously established as causal or associated with CSVD. Seven genes (*TTN, TENM4, COL7A1, MMP9, HMCN1, LAMA1,* and *TNC*) showed significant associations with CSVD compared to controls (Table 5).

**Table 5:** Significant genes identified from burden testing of rare, deleterious variants in genes identified in GO: biological process-enriched pathways

Gene	Variant carriers in cases (% of cohort)	Variant carriers in controls (% of cohort)	*P* value (adjusted)	Odds ratio
*COL7A1*, n (%)	6 (5.3)	4 (0.4)	0.000567 (0.03377)	10.646
*HMCN1*, n (%)	4 (3.5)	4 (0.4)	0.000838 (0.03663)	11.424
*LAMA1*, n (%)	8 (7.1)	14 (1.4)	0.000999 (0.03663)	4.863
*MMP9*, n (%)	4 (3.5)	3 (0.3)	0.000614 (0.03377)	14.743
*TENM4*, n (%)	6 (5.3)	9 (0.9)	0.000244 (0.02684)	7.478
*TNC*, n (%)	4 (3.5)	3 (0.3)	0.00157 (0.04934)	11.804
*TTN*, n (%)	14 (12.4)	31 (3.0)	0.0000277 (0.006094)	4.354

Burden analysis was performed using logistic regression with age and sex included as covariates. A nominal significance threshold of *P* = 0.05 after multiple testing correct was set

**Fig. 4:**
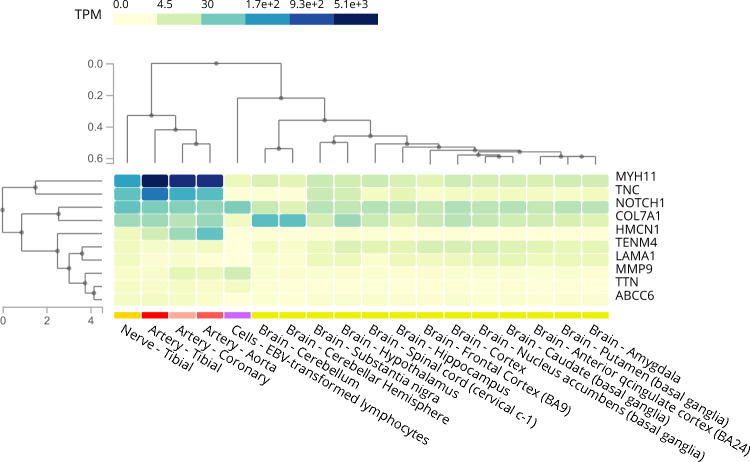
Gene expression map of vasculature and brain tissues for significant genes identified from PanelApp gene panels and gene set enrichment analysis

Gene expression analysis of identified genes was performed using GTEx and CELLxGENE. GTEx identified *MYH11, TNC, NOTCH1, COL7A1,* and *HMCN1* as highly expressed in arterial tissues, whilst *NOTCH1* had the highest expression across brain tissues amongst the genes tested (Fig. 4). Single-cell RNA expression data from CELLxGENE revealed *TENM4* had the highest expression within cerebral tissues, specifically neurons, whilst *TNC* had the highest expression within glial cell types, particularly within glioblasts and immature astrocytes. *MYH11* showed the highest expression across vascular cell types, particularly in vascular smooth muscle cells and perivascular cells*,* whilst both *MYH11* and *TNC* showed high expression in pericytes (Fig S1). Detailed information on all genes included in burden testing is provided in Table S9 and individual variants identified in significant genes are provided in Table S10.

## Discussion

In a cohort of 117 cases and 1,035 controls, we investigated potential disease-causing variants and gene-based associations with monogenic CSVD. A total of 18 patients (15.4%) carried a candidate disease-causing variant in a known or recently associated CSVD gene. Additionally, we identified a significant burden of rare, deleterious variants in ten genes within this cohort, including *ABCC6, NOTCH1, MYH11, TTN, TENM4, TNC, MMP9, HMCN1, LAMA1,* and *COL7A1* (Table 6).

**Table 6:** Gene expression, function, and relevance to CSVD for implicated genes

Gene	Function	Gene expression	Potential relevance to CSVD
*ABCC6*	ATP-binding cassette transporter regulating extracellular pyrophosphate, preventing ectopic mineralisation	Liver, kidney, small intestine, pancreas	Pathogenic variants cause pseudoxanthoma elasticum which has established cerebrovascular involvement, including ischaemic stroke, white matter disease, and cognitive decline [30–33]
*MYH11*	Myosin heavy chain subunit encoding smooth muscle myosin	Colon, blood vessels, urinary bladder, smooth muscle	Pathogenic variants cause non-syndromic thoracic aortic aneurysm and dissection through vascular smooth muscle dysfunction, which is also seen in cerebrovascular disease [34]. Several studies have identified cerebral angiopathy phenotypes associated with variants [35, 36]
*NOTCH1*	Transmembrane receptor regulating cellular development and differentiation, particularly arterial endothelial cells	Skin, ubiquitous across tissues	Paralogue of *NOTCH3* which causes CADASIL*.* Variants have been associated with a calcifying microangiopathy with leukoencephalopathy [37–39]
*COL7A1*	Encodes the α−1 chain of type VII collagen which is a structural component of the extracellular matrix and basement membrane	Skin, sex organs, cerebellum	Variants are associated with Chiari malformation type 1 [40]. *COL4A1/A2* subunits are known to cause monogenic CSVDs
*HMCN1*	Secreted glycoprotein that plays an important role in cell migration and cell–cell conjunction. Also regulates TGF-β activation and signal transduction	Blood vessels, lung	Variants are associated with childhood-onset essential hypertension as well as age-related macular degeneration, a disorder affecting the retinal vasculature [41]
*LAMA1*	Encodes laminin α−1, an essential glycoprotein component of the basement membrane involved in cell attachment, proliferation, and differentiation	Testes, kidney, cerebral tissues	Pathogenic variants cause Poretti–Boltshauser syndrome, which is characterised by cerebellar dysplasia and cysts, as well as retinal dystrophy which is observed in several monogenic CSVDs [42, 43]
*MMP9*	A matrix metalloproteinase involved in degradation of the extracellular matrix	Bone marrow & lymphoid tissues	Associated with atherosclerosis, Kawasaki disease, ischaemic stroke, Alzheimer’s disease, and BBB disruption and white matter injury [44, 45]
*TENM4*	Transmembrane protein regulating oligodendrocyte maturation and myelination of the central nervous system	Ovaries, glandular tissues, cerebral tissues	Pathogenic variants cause hereditary essential tremor which has been observed in several monogenic CSVDs [46, 47]
*TNC*	Matricellular glycoprotein involved in regulation of cellular development and tissue morphogenesis	Blood vessels, smooth muscle	Associated with neuroinflammation in stroke, cerebral vasospasm, and atherosclerosis aortic aneurysm [48]
*TTN*	Encodes titin, a large sarcomeric protein involved in muscle assembly, force transmission, and maintenance of resting tension	Heart/skeletal muscle	Pathogenic variants cause cardiovascular disorders including several subtypes of cardiomyopathy and myopathy [49]. Shown to regulate arterial stiffness through vascular smooth muscle cell tone [50]

Genes identified as significantly associated with CSVD through rare variant burden testing are summarised with respect to known or hypothesised molecular function, primary tissue and cell type expression based on the Human Protein Atlas, and proposed mechanistic or clinical relevance to cerebrovascular pathology. CSVD cerebral small vessel disease; TGF-β transforming growth factor beta; BBB blood–brain barrier

Five heterozygous variants were identified in *ABCC6* which causes pseudoxanthoma elasticum (PXE), an autosomal recessive connective tissue disorder characterised by fragile vasculature, skin laxity, and retinal haemorrhage [51, 52]. Two of these variants were previously identified as pathogenic in a recessive inheritance pattern; however, monoallelic variants are associated with a milder form of disease known as PXE forme fruste. Heterozygous variants in *ABCC6* have been associated with prominent PXE-related cardiovascular phenotypes and an increased risk of ischaemic stroke [30, 53, 54]. A significant burden of both rare and rare, predicted deleterious heterozygous variants was identified in *ABCC6*, highlighting its role as a potential cause of CSVD in this cohort.

Analysis of genes causal of disorders sharing CSVD symptomology identified a significant burden of rare, disease-causing variants in *NOTCH1* and *MYH11*. Three variants were detected in *NOTCH1*, a transmembrane receptor essential for vascular mural cell development [55]. Whilst traditionally associated with congenital disorders such as Adams–Oliver disease through a loss-of-function mechanism, gain-of-function variants have been identified to cause a chronic CNS disorder with calcifying microangiopathy and leukoencephalopathy [37–39, 56]. All reported pathogenic variants for this CNS disorder localise to the negative regulatory region (NRR), whereas variants in this study were identified in the epidermal growth factor-like repeat (EGFr) domains, highlighting the need for functional studies to determine potential converging mechanisms of pathogenesis.

*MYH11* encodes a myosin heavy chain subunit and is associated with non-syndromic thoracic aortic aneurysm and dissection (TAAD) [34]. Three candidate variants were identified in our case cohort, including one variant (p.Arg1765Gln) identified in DGR22 which has previously been classified as likely pathogenic for TAAD. Several cases of cerebral vascular pathologies caused by variants in *MYH11* have been identified, particularly as a cause of recurrent stroke in paediatric and young adult populations [35, 36]. The established role of *MYH11* variants in vascular smooth muscle dysfunction and its association with vascular arteriopathy suggest that cerebral manifestations may also fall within the phenotypic spectrum of *MYH11*-related disorders [57].

Two pathogenic variants were also identified in three patients in *SQSTM1,* which is causative of frontotemporal dementia and/or amyotrophic lateral sclerosis (FTD-ALS) [58]. FTD-ALS shares clinical signs and symptoms with CSVD including gait disturbance, cognitive decline, and white matter changes, and several studies have identified clinical signs of ALS in patients with genetically diagnosed CVSD [59, 60]. An additional pathogenic nonsense variant was identified in *ANGPTL6* which is causative of familial intracranial aneurysm (fIA) [61]. This variant has previously been identified in one patient with fIA and has been shown to result in complete loss of secretion of ANGPTL6 in cells [62, 63].

Our burden analyses further identified significant associations between CSVD and seven additional genes: *TTN, TENM4, COL7A1, MMP9, HMCN1, LAMA1*, and *TNC*. The significant association identified with *LAMA1* implicates the ECM laminin complex in CSVD pathogenesis [64]. *LAMA1, LAMB1,* and *LAMC1* each encode subunits of the laminin complex, a core structural component of the vascular basement membrane. Biallelic variants in *LAMA1* cause Poretti-Boltshauser syndrome, characterised by cerebellar dysplasia and retinal dystrophy, whilst protein-truncating variants in *LAMB1* have been reported to cause monogenic CSVD with hippocampal memory deficits and leukoencephalopathy*. LAMC1* has recently been associated with familial CSVD in an independent cohort, with one variant (p.Pro321Ser) identified in both studies [65]. Together, LAMA1, LAMB1, and LAMC1 form the heterotrimeric glycoprotein laminin 111 which self-organises with collagen IV, of which *COL4A1* and *COL4A2* are established monogenic CSVD genes, highlighting a convergent role for basement membrane dysregulation in CSVD pathogenesis [66, 67].

Several other key matrisomal genes were identified as significant in this study. *COL7A1* encodes the α1-subunit of collagen VII where pathogenic variants cause several forms of epidermolysis bullosa, an ECM disorder resulting in subepidermal blistering. Variants in *COL7A1* have also been associated with neurological disorders such as Chiari malformation [40, 68]. *MMP9* encodes a matrix metalloproteinase (MMP) that regulates ECM remodelling and has been implicated in blood–brain barrier disruption, ischaemic stroke, and white matter injury [44, 69]. *HMCN1* is a fibulin family ECM protein involved in cell adhesion and is associated with age-related macular degeneration [41]. It is also highly expressed in the vascular smooth muscle cells of the cerebral arteries, a key cell type dysregulated in monogenic CSVD [70]. *TNC* is an ECM glycoprotein expressed during vascular injury, where plasma protein levels of TNC have been associated with WMH in CSVD remodelling [48, 71]. The identification of multiple significant matrisomal protein-encoding genes highlights the role of ECM dysregulation in CSVD pathogenesis and the need for further characterisation as to their mechanistic role in these diseases.

*TENM4*, encoding teneurin transmembrane protein 4, is involved in oligodendrocyte development and central nervous system myelination, and is known to cause hereditary essential tremor [46]. Mutations in this gene have also been associated with an increased risk of Parkinson’s disease [47]. The high expression in neural tissues and role in myelination provides a plausible mechanism for *TENM4* as causative of white matter abnormalities and gait disturbance which is often seen in monogenic CSVD. *TTN* encodes the sarcomeric protein titin which is a critical structural component of striated muscle [72]. *TTN* is known to cause a spectrum of cardiac myopathies, including both dilated and hypertrophic cardiomyopathy, and is primarily expressed in skeletal and cardiac muscle [73]. Titin plays a role in regulating arterial stiffness through differential expression of titin isoforms in VSMCs, potentially implicating a role in CVSD through dysregulation of vascular compliance [74].

Whilst small sample sizes preclude genotype–phenotype analyses, phenotypic patterns were observed across variant carrier groups (Table S11). WMH were the most prevalent MRI finding across all groups. *TTN* variant carriers represented the largest subgroup (n = 14) and showed the highest rates of WMH (79%), ischaemic stroke (43%), and migraine (36%). *LAMA1* variant carriers (*n* = 8) showed universal WMH alongside ischaemic stroke and ocular abnormalities, whilst *NOTCH1* variant carriers presented predominantly with WMH, ischaemic stroke, and cognitive decline. *MMP9* variant carriers demonstrated the youngest mean age at referral (37.8 ± 14.4 years). For more comprehensive genotype–phenotype characterisation, prospective studies are required with standardised clinical and neuroimaging assessment.

In this study, we used a large cohort of clinically suspected CSVD patients, alongside a large control cohort free of neurological disease or dysfunction at an advanced age, to detect disease associations. Comprehensive clinical data were unavailable for a subset of the case cohort, and detailed clinical and neuroimaging data were not available for controls. Consequently, we cannot exclude the presence of subclinical CSVD findings in the control population, nor perform robust genotype–phenotype analyses for identified variants. Additionally, segregation analysis of candidate variants was unable to be performed due to an absence of consented family members. The associations identified here are not confirmed as causal, and the identified variants may play modifier roles in CSVD caused by alternate genetic variation or be causal of alternate disorders with phenotypic overlap with CSVD. Further, we cannot exclude that patients within this cohort represent early or atypical sporadic CSVD or alternative disorders which mimic CSVD. Whole-genome sequencing may also improve our ability to detect variants outside the coding regions which may play a role in CSVD. Validation in independent datasets and functional studies using relevant cell lines or animal models is required to confirm the role of implicated genes in CSVD pathogenesis.

## Conclusion

This study identified 18 rare, likely disease-causing variants in eight genes implicated in CSVD, including a significant burden of both rare and predicted deleterious variants in *ABCC6*. Pathogenic variants were also detected in four patients for neurological conditions with overlap to monogenic CSVD, including frontotemporal dementia with amyotrophic lateral sclerosis and familial intracranial aneurysm. Two genes previously implicated in conditions with phenotypic overlap to CSVD (*NOTCH1, MYH11*) were also significantly associated with CSVD in this cohort. Finally, rare, likely deleterious variants in *TTN, TENM4*, *TNC, COL7A1, MMP9, HMCN1,* and *LAMA1* were found to be significantly enriched in the case cohort, suggesting a potential contributory or causal role in CSVD. Notably, several of these genes are involved in the matrisome, further implicating its role in the molecular pathogenesis of CSVD.

## Supplementary Information

Below is the link to the electronic supplementary material.

Supplementary file1 (PNG 3327 KB)

Supplementary file2 (XLSX 59 KB)

## Data Availability

The datasets used within the current study are available from the corresponding author upon reasonable request.
