# Supplementary figures and images for: Whole-exome sequencing identifies matrisomal gene associations in monogenic cerebral small vessel disease
Source: J Neurol. 2026 Jul 21;273(8):478. doi: 10.1007/s00415-026-14018-2 (PMC13388773; doi:10.1007/s00415-026-14018-2)

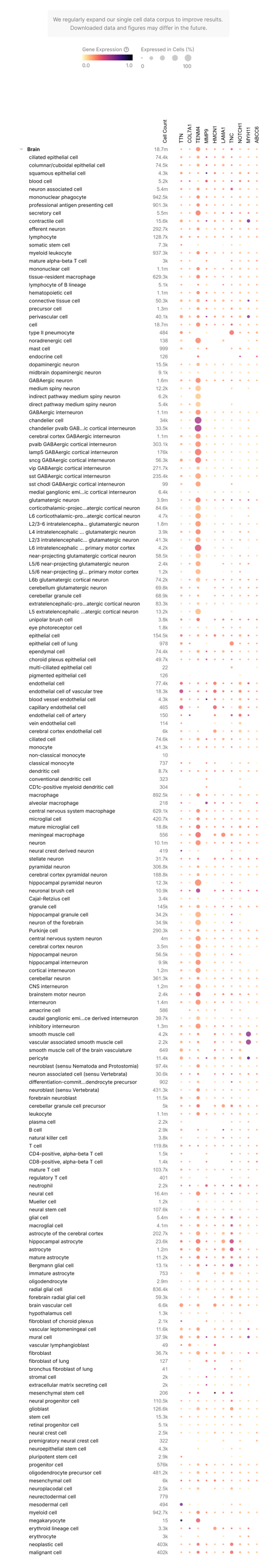

Supplement: Supplementary file 1 — Supplementary file1 (PNG 3327 KB) [file 415_2026_14018_MOESM1_ESM.png]
